# Effects of Electromagnetic Waves with LTE and 5G Bandwidth on the Skin Pigmentation In Vitro

**DOI:** 10.3390/ijms22010170

**Published:** 2020-12-26

**Authors:** Kyuri Kim, Young Seung Lee, Nam Kim, Hyung-Do Choi, Dong-Jun Kang, Hak Rim Kim, Kyung-Min Lim

**Affiliations:** 1 College of Pharmacy, Ewha Womans University, Seodaemungu, Seoul 03760, Korea; kyuri@ewhain.net; 2 Radio & Satellite Research Division, Electronics and Telecommunications Research Institute, Yuseong-gu, Daejeon 34129, Korea; lys009@etri.re.kr (Y.S.L.); choihd@etri.re.kr (H.-D.C.); 3 Department of Computer and Communication Engineering, Chungbuk National University, Seowon-gu, Cheongju 28644, Korea; namkim@chungbuk.ac.kr; 4 Department of Pharmacology, College of Medicine, Dankook University, Cheonan, Chungnam 31116, Korea; kdu1313@naver.com (D.-J.K.); hrkim@dankook.ac.kr (H.R.K.)

**Keywords:** skin pigmentation, melanogenesis, artificial human epidermis, electromagnetic waves, LTE, 5G

## Abstract

With the rapid growth of wireless communication devices, the influences of electromagnetic fields (EMF) on human health are gathering increasing attention. Since the skin is the largest organ of the body and is located at the outermost layer, it is considered a major target for the health effects of EMF. Skin pigmentation represents one of the most frequent symptoms caused by various non-ionizing radiations, including ultraviolet radiation, blue light, infrared, and extremely low frequency (ELF). Here, we investigated the effects of EMFs with long-term evolution (LTE, 1.762 GHz) and 5G (28 GHz) bandwidth on skin pigmentation in vitro. Murine and Human melanoma cells (B16F10 and MNT-1) were exposed to either LTE or 5G for 4 h per day, which is considered the upper bound of average smartphone use time. It was shown that neither LTE nor 5G exposure induced significant effects on cell viability or pigmentation. The dendrites of MNT-1 were neither lengthened nor regressed after EMF exposure. Skin pigmentation effects of EMFs were further examined in the human keratinocyte cell line (MNT-1-HaCaT) co-culture system, which confirmed the absence of significant hyper-pigmentation effects of LTE and 5G EMFs. Lastly, MelanoDerm™, a 3D pigmented human epidermis model, was irradiated with LTE (1.762 GHz) or 5G (28 GHz), and image analysis and special staining were performed. No changes in the brightness of MelanoDerm™ tissues were observed in LTE- or 5G-exposed tissues, except for only minimal changes in the size of melanocytes. Collectively, these results imply that exposure to LTE and 5G EMFs may not affect melanin synthesis or skin pigmentation under normal smartphone use condition.

## 1. Introduction

Artificial electromagnetic fields (EMFs) were discovered in 1887 by German physicist Heinrich Hertz. Since then, communication technology has advanced remarkably and numerous electronic communication devices have been developed, which have become an everyday necessity [1]. Electromagnetic waves can be classified into extremely low frequency (ELF), radio frequency (RF) radiation, and microwave radiation, according to the frequency range [2]. In general, the range of radio frequency (RF) that is widely used in real life is 3 kHz to 300 GHz. Humans are extensively exposed to RF via various wireless communications, including Wi-Fi and mobile phones [1,3,4]. Recently, the communications industry has been resorting to the sub-THz frequency spectrum to fulfill the need for faster data transmission. The fifth-generation network, 5G, which uses the frequencies from 28 GHz up to 60 GHz (FCC Report 16-89), has been very recently introduced to mobile communication devices. The industry has assumed that there would be no human health risk associated with 5G frequency and has extended the application of 5G to heterogeneous networks that integrate 4G, Wi-Fi, and millimeter waves (mmW), as well as to other wireless devices [5]. Although active plans for the use of 5G have been established, concerns about non-thermal biological effects caused by electromagnetic radiation or potential adverse health outcomes are also escalating [6]. Some reports have suggested a moratorium or limitation on the deployment of 5G because its way of interacting with the human body has changed from direct absorption to a more complex way. Thus, studies on the effects of 5G electromagnetic waves on human health are in increasing demand [7].

According to previous studies, EMFs may have effects on several cell functions, including cell migration, differentiation, death, and cell stress [8]. In addition, exposure to electromagnetic waves has been reported to be associated with many harmful effects, such as genetic damage, neurological diseases, reproductive disorders, immune disorders, kidney damage, electromagnetic hypersensitivity, and leukemia [9,10,11,12,13,14]. The skin is a barrier of the body against various external harmful factors. Since the skin is located at the outermost layer of the body, it is easily exposed to chemical substances, physical stimuli, and environmental contaminants, including ultraviolet rays, electromagnetic waves, radiation, and heat [15,16]. Upon exposure to harmful stresses, the skin responds in various ways, such as forming wrinkles, losing moisture, developing skin irritation or skin sensitization responses, or hyper- or hypopigmentation. In particular, hyperpigmentation, i.e., abnormal melanin synthesis, is a well-established response to non-ionizing radiations such as UV-A, UV-B, blue light, and near infrared [17]. 

Hyperpigmentation is manifested by excessive melanin production in melanocytes, which can be found in diverse organs including skin, hair follicles, eyes, or inner ear [18,19]. Melanin synthesis requires a number of specific enzymes and structural proteins. In particular, tyrosinase (TYR) is a critical enzyme for the synthesis of melanin. Furthermore, the expression of other melanogenic markers, such as tyrosinase-related protein 1 (TRP-1), tyrosinase-related protein 2 (TRP-2), and microphthalmia-associated transcription factor (MITF), are known to affect melanin synthesis in melanocytes [20]. Of note, in a recent study, it was found that pulsed electromagnetic fields (PEMFs) (60 Hz) induced skin pigmentation in zebrafish by increasing the activity of TRP-1, mediated by the phosphorylation of ERK and p38 [21], reflecting that melanin synthesis and melanocytes may be affected by EMFs [22]. However, it is not yet known whether 5G or even long-term evolution (LTE) EMFs could affect melanogenesis, to the best of our knowledge.

**Scheme 1:**
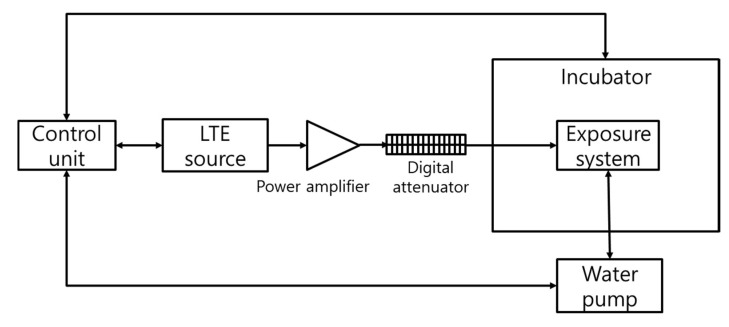
Diagram of LTE exposure device.

**Scheme 2:**
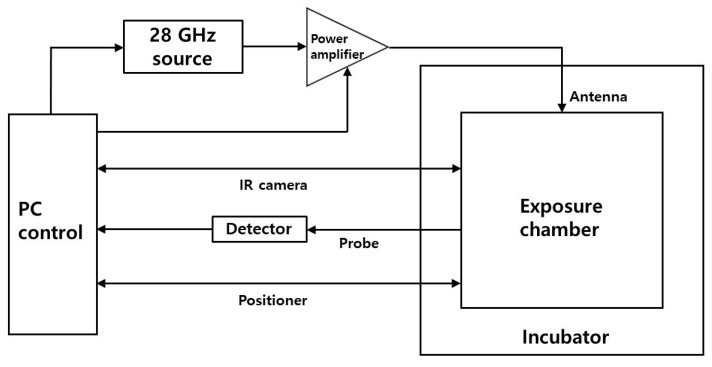
Diagram of 5G exposure system.

Here, using our LTE and 5G cell exposure systems (Scheme 1 and Scheme 2), we examined the effects of LTE and 5G EMFs on skin pigmentation in vitro [23]. Especially, the 5G in vitro “all-in-one” exposure system for 28 GHz frequency, which is one of the important millimeter-wave frequencies for 5G commercial services, has been developed [24,25]. Human and murine melanoma cell lines (MNT-1 and B16F10), human keratinocyte cell line (MNT-1- HaCaT) co-culture, and a 3D pigmented human epidermis model (MelanoDerm™, MatTek, Ashland, MA, USA) were employed to examine the pigmentary effects of LTE and 5G EMFs with the exposure time of EMF set to 4 h per day, which is known to be the upper bound of smartphone usage time per day [26].

## 2. Results

### 2.1. Effect of EMF on Cell Viability

**Figure 1:**
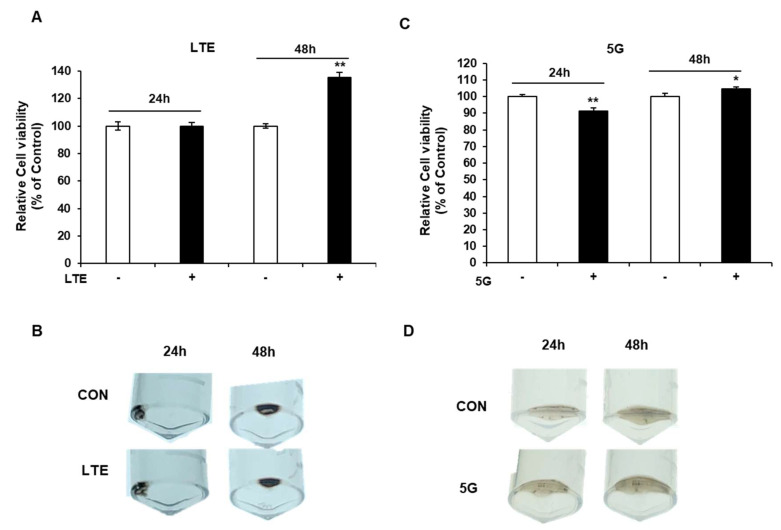
Effects of long-term evolution (LTE) and 5G on cell viability and melanin synthesis. The murine melanoma cell line, B16F10, or human melanoma cell line, MNT-1, were exposed to either LTE (1.762 GHz) or 5G (28 GHz) for 4 h per day for 2 days. (**A**,**B**) Cell viability was determined by 4-[3-(indophenyl)-2-(4-nitrophenyl)-2H-5-tetrazolio]-1,3-benzene disulfonate (WST-1) assay or 3-(4,5-dimethylthiazol-2-yl)-2,5- diphenyltetrazolium bromide (MTT) assay. (**C**,**D**) Macroscopic view of MNT-1 cell pellets observed after 4 h exposure of LTE or 5G. Data are presented with the mean ± SE *(N* = 3~5). * *p* < 0.05, ** *p* < 0.01.

First, the effects of LTE and 5G EMFs on cell viability were examined in B16F10, a murine melanoma cell line. After the exposure of LTE (1.762 GHz) at an intensity of 8 W/kg (Figure 1A) or 5G (28 GHz) at an intensity of 10 W/m^2^ (Figure 1C) for 4 h per day for 2 days, cell viability was measured with formazan-based assays, 3-(4,5-dimethylthiazol-2-yl)-2,5-diphenyltetrazolium bromide (MTT) and 4-[3-(indophenyl)-2-(4-nitrophenyl)-2H-5-tetrazolio]-1,3-benzene disulfonate (WST-1). As a result, it was confirmed that both LTE and 5G EMFs were not cytotoxic to B16F10. Human melanoma cells, MNT-1, were used to establish the effect of LTE and 5G EMFs on skin pigmentation. As shown in Figure 1B,D, there was no evident change in pellet color of MNT-1 cells after exposure to LTE or 5G, suggesting that LTE or 5G, at this condition, did not induce significant effects on skin pigmentation.

### 2.2. Effects of EMF on Cell Morphology of Human Melanoma Cell, MNT-1 Cell

**Figure 2:**
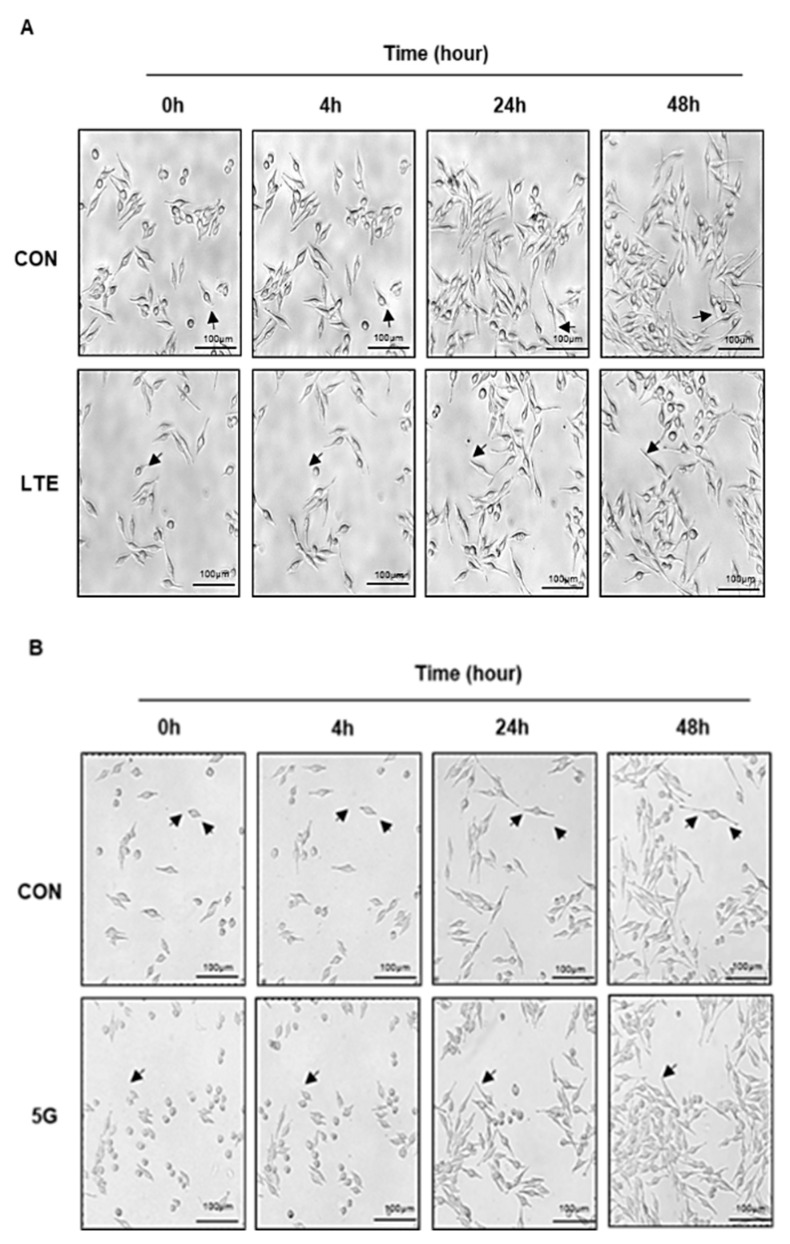
Effects of electromagnetic fields (EMFs) on the dendrite formation in MNT-1 cell lines. MNT-1 cells were exposed to either LTE (1.762 GHz) or 5G (28 GHz) for 4 h per day on indicated days. Morphological changes were observed under optical microscopy (100×). (**A**) Dendrite formation by MNT-1 cells after LTE (1.762 GHz) exposure. (**B**) Dendrite formation by MNT-1 cells after 5G (28 GHz) exposure. Black arrows indicated the representative dendrite formation as the cultivation time continues. Scale bars represent 100 μm.

Melanosomes are transferred through the dendrites of melanocytes to keratinocytes during melanogenesis [27]. Thus, the morphological change of melanoma cells can be used as an indicator to evaluate the hyperpigmentary effect of EMFs [28]. MNT-1 cells were seeded on an 8-well chamber slide with a cell location grid, and the cells at the same location were microscopically photographed at the indicated time. Cells were exposed to either LTE or 5G for 4 h per day. As the result, the dendrites of MNT-1 cells were not regressed or extended after LTE or 5G exposure (Figure 2A,B). In addition, when judged by visual inspection, the number of cells in LTE- or 5G-irradiated group was not diminished, supporting that neither LTE nor 5G inflicted toxicity in our experimental condition.

### 2.3. Effect of EMF on Cell Morphology in Keratinocyte-Melanocyte Co-Culture

**Figure 3:**
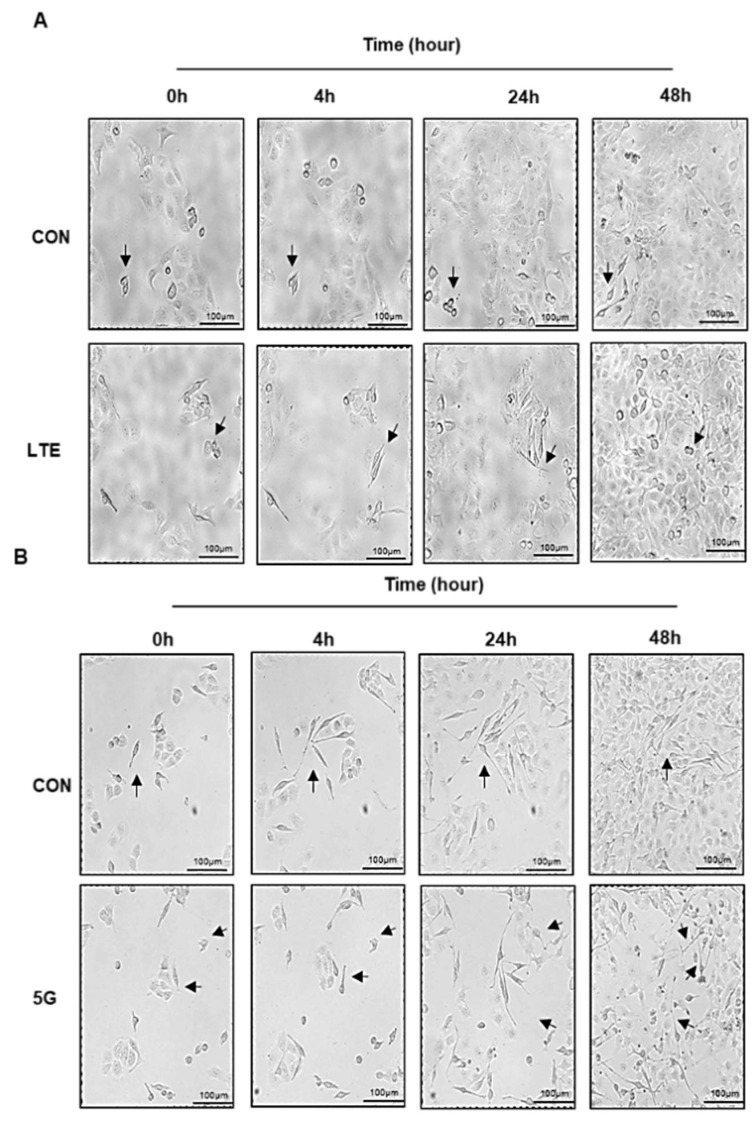
Effects of EMFs on the dendrite formation in co-cultured cell lines. MNT-1 and HaCaT cells were co-cultured in 8-well chamber and then exposed to either LTE or 5G for 4 h per day. (**A**) Dendrite formation changes on MNT-1/HaCaT co-culture cells after LTE exposure. (**B**) Dendrite formation changes on co-culture cells after 5G exposure. Black arrows indicated the representative dendrite formation as the cultivation time continues.

Skin pigmentation is mostly initiated by the intricate cellular and molecular interactions between melanocytes and keratinocytes, which are the main cellular components of the epidermis [17]. To examine the effects of EMFs on keratinocyte–melanocyte interaction, the melanoma cell line MNT-1 and the human keratinocyte cell line HaCaT were co-cultured in order to mimic in vivo situation. There was a slight change in the dendrites of MNT after 2 days of LTE or 5G EMF exposure (4 h per day), but pigmentation was not or at most mildly affected (Figure 3A,B).

### 2.4. Effect of EMF on mRNA Level of Melanogenic Enzymes

**Figure 4:**
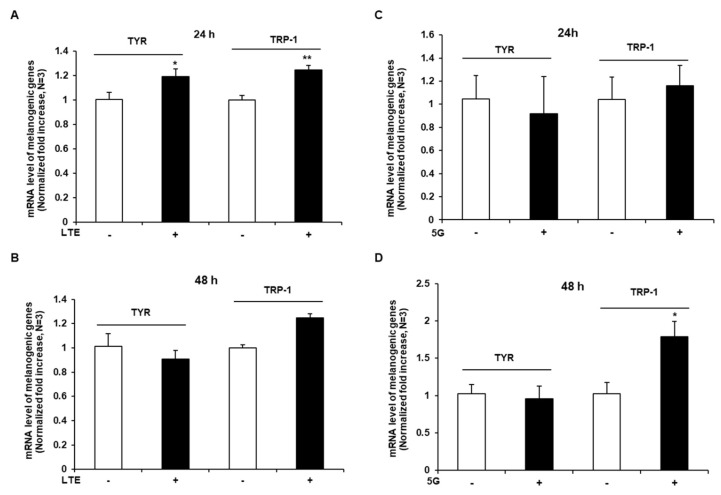
Effects of EMFs on mRNA level of melanogenic genes. The cells were exposed to either LTE or 5G for 4 h per day on indicated days. mRNA expression levels of tyrosinase and tyrosinase-related protein 1 (TRP-1) after (**A**) 24 h, and (**B**) 48 h exposure of LTE (1.762 GHz). mRNA expression levels of tyrosinase and TRP-1 after (**C**) 24 h, and (**D**) 48 h exposure of 5G (28 GHz). * *p* < 0.05, ** *p* < 0.01.

Tyrosinase (TYR) and tyrosinase-related protein 1 (TRP-1) are crucial melanogenic enzymes in melanin synthesis. Here, murine melanoma cells, B16F10, were exposed to either LTE or 5G for 4 h per day. Then, 24 or 48 h after the exposure, the cells were lysed using Trizol, and mRNA expression levels of TYR and TRP-1 were determined using real-time polymerase chain reaction (real-time-PCR). As a result, significant but only slight increases in the expression of TYR and TRP-1 were observed (Figure 4).

### 2.5. Effect of LTE and 5G on a Pigmented Human Skin Model, MelanoDerm™

**Figure 5:**
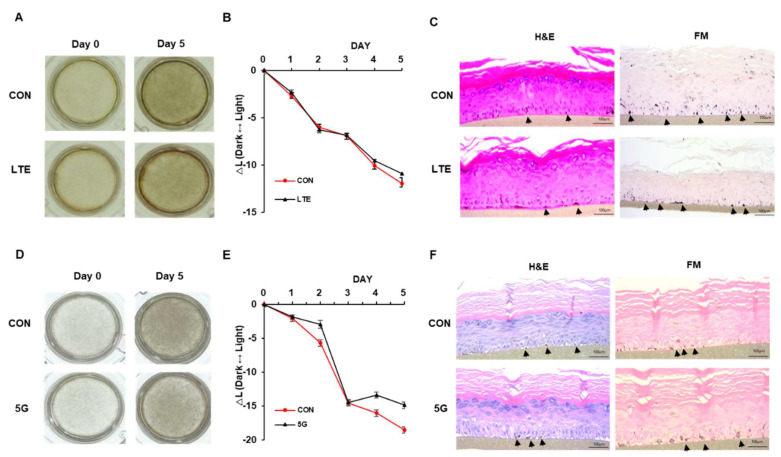
Effects of EMFs on MelanoDerm™, an artificial pigmented skin model. (**A**) Color of LTE-exposed 3D human skin model (MelanoDerm™; MatTek). (**B**) ΔL value in skin tissue between baseline and 5 days after LTE exposure. (**C**) Hematoxylin and eosin (H&E)- and Fontana–Masson (FM)-stained tissue after LTE exposure. (**D**) Color of 5G-exposed MelanoDerm^TM^. (**E**) ΔL value in skin tissue between baseline and 5 days after 5G exposure. (**F**) Hematoxylin and eosin (H&E) and Fontana–Masson (FM)-stained tissues after 5G exposure. Black arrow heads indicated the stained melanocytes.

MelanoDerm™, a pigmented human skin model, exhibits in-vivo-like morphological characteristics of human epidermis and has been widely used to study the physiological mechanisms of skin pigmentation. Normally, MelanoDerm™ becomes increasingly pigmented as the cultivation days continue. After continuous exposure to LTE at 8 W/kg for 5 days, there was no difference in visual evaluation (Figure 5A) and image analysis for brightness (Figure 5B). The tissues were stained with hematoxylin and eosin (H&E) and Fontana–Masson (FM) melanin stain on the last day of the experiment (Figure 5C), which confirmed that there was little difference between the control and the LTE-exposed sample. To confirm the effect of 5G on skin pigmentation, MelanoDerm™ was exposed to 5G at 10 W/m^2^ for 5 days. The results revealed that there were no significant changes in pigmentation in visual evaluation (Figure 5D) and image analysis for brightness (Figure 5E). H&E and FM staining also showed that the quantity of melanin was not increased (Figure 5F).

## 3. Discussion

The fifth-generation network, 5G, boasts a high-speed data rate, low latency, high mobility, high energy efficiency, and high traffic density. As the development and commercial operation of the 5G network is speeding up, 5G not only provides faster internet, but is also utilized in various medical devices [29]. However, the deployment of 5G has raised public concern due to insufficient evidence that can exclude the risk to human health and the environment [30]. The previous report suggested that millimeter waves (mmW) can increase skin temperature, induce genetic modification, and promote the synthesis of a specific protein, which is related to oxidative stress, inflammation, and metabolic processes [7]. In particular, exposure to mmW (60.4 GHz) with an incident power density of 20 mW/cm^2^ in human skin cells has been reported to alter endoplasmic reticulum function as well as expression of genes involved in cellular communication and homeostasis of endoplasmic reticulum [31,32]. In addition, it was reported that the depth of skin penetration of EMFs rapidly decreased as the frequency increased, resulting in more than 90 percent of the transmitted electromagnetic power being absorbed in the epidermis and dermal layers [33], reflecting that the skin might be the major target of 5G irradiation.

Although studies on radio frequency (RF) radiation and related health effects have been published over the years, the results are controversial. According to a previous study, it has been suggested that when the skin is exposed to electromagnetic waves, epidermal layer damage or skin cancer may occur [34,35]. Changes in the expression of genes and metabolites by electromagnetic waves have also been reported [36,37,38]. More specifically, RF EMFs can induce oxidative stress and oxidative DNA base damage [7,39,40]. After skin was exposed to 900 MHz radio waves emitted by a mobile phone, lipid peroxidation (LPO) increased due to oxidative stress, or a change in antioxidant activity was confirmed [41,42,43]. ELF EMFs have also been reported to be able to interact with biological systems. In particular, ELF EMFs are involved in many types of cell processes, including cell migration, differentiation, apoptosis, and stress responses [8,44]. Furthermore, a recent study has documented the possibility of ultra-low-frequency electric fields to induce skin pigmentation [45]. However, RFs are widely employed in medical devices used for skin tightening and improvement of skin laxity and cellulite without any significant side effects [46,47].

Our study suggests that LTE or 5G may not affect skin pigmentation or cell viability of melanocytes. The exposure of LTE or 5G EMFs on murine melanoma cells, B16F10, did not induce cytotoxicity. The absence of any effects of LTE or 5G on skin pigmentation was confirmed at multiple levels ranging from MNT-1 cell monolayer culture to MNT-1 and HaCaT co-culture and a 3D pigmented human skin model with respect to melanin content, brightness, and morphology. In these assay systems, neither LTE nor 5G EMFs induced any characteristics of melanogenesis following a 4 h-per-day exposure scheme, which is the upper bound of average smartphone use time in Korea [26]. Therefore, it could be inferred that under the normal condition of smartphone use, skin pigmentation effects of LTE or 5G appear unlikely to occur.

Interestingly, while macroscopic skin pigmentation was not observed, mRNA levels of TYR and TRP-1 were slightly increased by LTE and 5G, suggesting that exposure to LTE or 5G with stronger intensity or for a prolonged time may affect skin pigmentation. In addition, in the presence of melanogenic stimuli that can occur together with RF exposure, such as ultraviolet radiation [48] or heat [49], it could not be excluded that LTE or 5G may potentiate skin pigmentation, although further studies are necessary to confirm this. Therefore, it would be interesting to examine the effects of LTE and 5G on skin pigmentation under more extreme exposure scenarios in the future.

In summary, we have shown that LTE (1.78 GHz) and 5G (28 GHz) did not strongly affect melanin synthesis or cell viability in human or murine melanoma cells or a 3D pigmented skin model. This indicates that human skin pigmentation may not be affected by exposure to LTE or 5G electromagnetic fields. However, this needs to be examined further using human primary melanocytes to shed more light on the potential effects of EMFs on skin and human health in general.

## 4. Materials and Methods

### 4.1. Materials and Reagents

The B16F10 cell line from C57BL/6 mice was purchased from ATCC (Manassas, VA, USA). Cells were maintained in standard culture conditions, Dulbecco’s Modified Eagle’s Medium (DMEM) supplemented with antibiotics (100 U/mL of penicillin A and 100 U/mL of streptomycin), and 10% fetal bovine serum (FBS) at 37 °C in a humidified atmosphere containing 5% CO_2_. At 80% cell confluence, adherent cells were detached with a solution of 0.05% trypsin (Hyclone, South Logan, UT, USA). MNT-1 cells were maintained in minimum essential medium supplemented with 10% DMEM, 20% fetal bovine serum (FBS), 1 M HEPES, and streptomycin-penicillin (100 U/mL each) at 37 °C in a humidified atmosphere containing 5% CO_2_. Monolayers of 80% confluence cells were cultured with 0.05% trypsin (Hyclone, South Logan, UT, USA).

### 4.2. Radio Frequency Exposure System

The LTE exposure system used in this study was the radial transmission line (RTL) exposure system. The LTE signal (1.762 GHz) was applied to the exposure system. This LTE exposure chamber is specially designed to control environmental conditions, including ventilation, humidity, and temperature. In order to maintain the CO_2_ density and humidity inside the chamber, the gas from the incubator flows through the entire chamber. The water pump works by circulating water through the entire cavity floor in order to protect temperature rise of the culture medium during LTE exposure [23].

### 4.3. 5G Exposure System

The 5G exposure system for 28 GHz experiments was developed for in vitro studies [25]. The “all-in-one” system integrates every system component into a single unit. The exposure chamber is designed based on the field uniformity [24]. Actual uniformity inside the chamber was checked based on planar measurements. Temperature regulation to suppress thermal effects during exposures can also be achieved by using the measured cell temperatures based on the infrared (IR) camera via real-time feedback, and controlling the air-flow rates of the incubator during high-power experiments. A personal computer (PC) can record and display all the experimental data during exposures. A graphical user interface (GUI) of the control software (SW) enables us to easily define all the operating conditions as well.

### 4.4. Cell Viability Assay

To identify cytotoxicity of both LTE and 5G exposure, murine melanoma cells were seeded into 48-well plates and then exposed to EMF for 4 h a day on indicated days. A 4-[3-(indophenyl)-2-(4-nitrophenyl)-2H-5-tetrazolio]-1,3-benzene disulfonate (WST-1) solution was used to investigate the cytotoxicity effect of LTE exposure. Additionally, 3-(4,5-dimethylthiazol-2-yl)-2,5- diphenyltetrazolium bromide (MTT) [50] was used to investigate the cytotoxicity effect of 5G exposure. B16F10 cells were incubated with 0.25 mL of 0.5 mg/mL 3-(4,5-dimethylthiazol-2-yl)-2,5-diphenyltetrazolium bromide (MTT) solution in DMEM for 2 h at 37 °C. Blue formazan dye was dissolved in 0.25 mL of DMSO for 30 min, and 200 μL of supernatant was measured as the absorption value at 540 nm. All measurements were performed in triplicate.

### 4.5. RNA Isolation

B16F10 cells were washed twice with phosphate-buffered saline after EMF exposure and were lysed using Trizol (Invitrogen, CA, USA). After the addition of chloroform, samples were centrifuged at 12,000 rpm for 10 min. The aqueous phase was mixed with isopropanol, and RNA pellets were collected by centrifugation (12,000 rpm, 15 min, 4 °C). RNA pellets were washed with 70% ethanol and dissolved in RNase-free, DEPC (diethyl pyrocarbonate)-treated water (Waltham, MA, USA). The RNA yield was estimated by determining the optical density at 260 nm with a NanoDrop 1000 spectrophotometer (NanoDrop Technologies, INC., Wilmington, DE, USA).

### 4.6. Real-Time Polymerase Chain Reaction (Real-Time PCR)

Relative expression levels of mRNAs were measured by quantitative real-time PCR. cDNA was synthesized from 1250 ng of total RNA with oligo(dT) (Bioelpis, Seoul, Korea). SYBR Green PCR master mix and a StepOnePlus^TM^ Real-time PCR machine (Applied Biosystems, Warrington, UK) were used in each reaction. The sequence of primers was as follows: forward tyrosinase, 5′-GGGCCC AAATTGTACAGAGA-3′; reverse tyrosinase, 5′-ATGGGTGTTG ACCATTGTT-3′; forward TRP-1, 5′-GTTCAATGGCCAGGTCAGGA-3′; reverse TRP-1, 5′- CAGACAAGAAGCAACCCCGA-3′. Cycling parameters were 50 °C for 2 min, 95 °C for 10 min, 40 cycles of 95 °C for 15 s, and 50 °C for 1 min.

### 4.7. MelanoDerm™, 3D Pigmented Human Epidermal Skin Model

MelanoDerm™ was purchased from MatTek Corporation (Ashland, MA, USA). Following its arrival, the skin model was incubated in a humidified 37 °C, 5%, CO_2_ incubator overnight (18–24 h) prior to the experiment. The skin model was exposed to an LTE (1.762 GHz) or 5G (28 GHz) signal for 5 days, while the negative control skin models were incubated in the incubator. Color alteration of each skin tissue was photographed every day for 5 days. The photograph of each skin tissue was analyzed by Adobe Photoshop CS6 software, and the L value was obtained from the Lab color model of the color picker. ΔL represents the average L value from five portions including up, down, left, right, and middle of the skin tissue. All measurements were performed in triplicate.

For the histological examination, all samples were fixed in 4% phosphate-buffered formalin (PFA) for 24 h with gentle shaking. Fixed samples were paraffin-embedded and cut into 5-mm sections using microtome (RM2335, Leica, Wetzlar, Germany). Hematoxylin and eosin (H&E) staining was performed one day after sectioning. For H&E staining, paraffin sections were deparaffinized and then hydrated in descending ethanol concentrations. Next, sections were stained with 0.1% Mayer’s hematoxylin for 10 min, and 0.5% eosin in 95% EtOH. After staining with H&E, the washing steps were immediately and sequentially performed as follows: dipped in distilled H2O until eosin stopped streaking, dipped in 50% EtOH 10 times, dipped in 70% EtOH 10 times, incubated in 95% EtOH for 30 s, and incubated in 100% EtOH for 1 min. Then, samples were covered with the mounting solution (6769007, Thermo Scientific, Waltham, MA, USA) and examined under the light microscope (BX43, OLYMPUS, Shinjuku, Japan).

For Fontana–Masson argentaffin staining, paraffin sections were incubated with ammoniacal silver nitrate for 1 h at 60 °C, followed by two washes with distilled water. For color development, samples were incubated with 0.2% working solution of gold chloride for 10 min and immediately rinsed 10 times with distilled water. After the final washes, samples were incubated with 5% sodium thiosulfate for 5 min to fix silver and rinsed with running water for 1 min. After silver fixation, nuclear fast red (60700, Fluka, Ronkonkoma, NY, USA) was used for counterstaining, and samples were rinsed with running water for 1 min.

### 4.8. Statistical Analysis

Results are expressed as the mean ± standard error of the mean (SEM) of three or more independent experiments. The statistical analyses were performed by Student’s t-test or two-way ANOVA. *p*-value < 0.05 was considered statistically significant.

## Data Availability

The data presented in this study are available on request from the corresponding author. The data are not publicly available since they are raw data.

## Abbreviations

EMFs	Electromagnetic fields
ELF	Extremely low frequency
RF	Radio frequency radiation
NIR	Non-ionizing radiation
LTE	Long-term evolution
mmW	Millimeter waves
TYR	Tyrosinase
TRP-1	Tyrosinase-related protein 1
TRP-2	Tyrosinase-related protein 2
MITF	Microphthalmia-associated transcription factor
PEMFs	Pulsed electromagnetic fields
MTT	(3-(4,5-dimethylthiazol-2-yl)-2,5- diphenyltetrazolium bromide)
WST-1	(4-[3-(indophenyl)-2-(4-nitrophenyl)-2H-5-tetrazolio]-1,3-benzene disulfonate)
H&E	Hematoxylin and eosin
FM	Fontana–Masson
